# Prevalence of Lung Cancer Screening Among Eligible Adults in 4 US States in 2021

**DOI:** 10.1001/jamanetworkopen.2023.19172

**Published:** 2023-06-21

**Authors:** Kristin G. Maki, Naomi Q. P. Tan, Iakovos Toumazis, Robert J. Volk

**Affiliations:** 1 Karmanos Cancer Institute, Wayne State University School of Medicine, Detroit, Michigan; 2 University of Texas MD Anderson Cancer Center, Houston

## Abstract

This cross-sectional study estimates the prevalence of lung cancer screening among eligible adults in 2021 and examines factors associated with screening.

## Introduction

Despite recommendations from the US Preventive Services Task Force (USPSTF) endorsing lung cancer screening (LCS), data from the 2019 Behavioral Risk Factor Surveillance System (BRFSS) showed that 12.8% of eligible adults received a computed tomographic (CT) scan to check for lung cancer.^1^ Screening rates declined during the COVID-19 pandemic.^2^ Increasing LCS among eligible adults is a national priority.^3^ We estimated LCS using data from the 2021 BRFSS and examined factors associated with LCS.

## Methods

The 2021 BRFSS^4^ data were collected from January 2021 through January 2022; 4 states (Maine, Michigan, New Jersey, and Rhode Island) are included the LCS module. We included adults aged 55 to 79 years with at least a 30-pack-year smoking history who currently smoked or quit within the past 15 years (eMethods in Supplement 1).^5^ We excluded respondents who reported a history of lung cancer. The study was exempt from institutional review board oversight and informed consent by the Common Rule given its use of publicly available data. We followed the STROBE reporting guideline.

We calculated the percentage and 95% CIs of eligible respondents who reported receiving a CT scan for LCS and conducted χ^2^ tests to assess statistically significant differences across demographic categories and subgroups based on lung cancer risk. We also examined factors associated with LCS using multivariable hierarchical logistic regression and reported odds ratios (ORs). We used the survey package in R, version 4.2.1 (The R Project) to account for the data design, with a 2-sided *P* < .05 considered significant.

## Results

The weighted sample included 112 399 respondents who underwent LCS (mean [SD] age, 65.7 [5.8] years); 49.5% were males and 50.5% were females and 86.7% were White. Prevalence of LCS was 21.2%, an increase of 8.4% from 2019.^1^ Respondents who underwent LCS reported a high pack-year smoking history (mean [SD], 57.3 [49.6] pack-years; 95% CI, 50.8-63.8 pack-years). The mean (SD) time since quitting was 6.9 (4.6) years (95% CI, 5.8-8.0 years) (Table 1). Of respondents who underwent LCS, 4.5% (95 CI, −4.5% to 13.5%) had no health insurance and 27.7% (95% CI, 16.0%-39.5%) reported poor health.

**Table 1.** Demographic and Lung Cancer Risk Factors Associated With LCS Eligibility and Completion of Screening

Characteristic	Respondents eligible for LCS		Respondents screened for LCS		*P* value^a^
	Weighted No.	Weighted % (95% CI)	Weighted No.	Weighted % within group (95% CI)	
Eligible for LCS January 2021-January 2022					
Not screened	418 393	78.8 (75.1 to 82.5)	NA	NA	<.001
Screened	112 399	21.2 (17.5 to 24.9)	112 399	21.2 (17.5 to 24.9)	
State					
Maine	52 002	9.5 (8.2 to 10.8)	10 221	20.0 (15.4 to 24.5)	.21
Michigan	299 670	55.0 (50.4 to 59.5)	67 200	22.6 (17.2 to 28.0)	
New Jersey	169 728	31.1 (26.4 to 35.8)	27 831	17.5 (10.9 to 24.1)	
Rhode Island	23 913	4.4 (3.5 to 5.2)	7148	30.3 (21.8 to 38.9)	
**Demographic characteristics**					
Sex					
Male	290 460	53.3 (48.7 to 57.9)	55 618	19.9 (15.1 to 24.6)	.46
Female	254 853	46.7 (42.1 to 51.3)	56 781	22.6 (17.0 to 28.3)	
Race and ethnicity^b^					
Black	24 247	4.6 (2.4 to 6.8)	3912	16.1 (2.2 to 30.0)	.09
Hispanic	15 089	2.9 (1.2 to 4.5)	2716	18.0 (−0.3 to 36.4)	
Multiracial	3087	0.6 (0.3 to 0.9)	510	16.6 (−5.1 to 38.3)	
White	467 654	89.1 (85.9 to 92.4)	95 286	21.0 (17.3% to 24.7)	
Other	14 516	2.8 (0.7 to 4.8)	7515	52.8 (16.0 to 89.5)	
Educational attainment					
Less than high school diploma	84 366	15.5 (11.6 to 19.3)	17 493	20.7 (9.9 to 31.5)	.33
High school diploma or GED	229 878	42.2 (37.5 to 46.8)	40 113	17.7 (11.9 to 23.4)	
Some college or technical school	172 585	31.7 (27.3 to 36.0)	39 296	24.1 (17.7 to 30.5)	
4 y college or more	58 118	10.7 (8.6 to 12.7)	15 131	27.0 (18.6 to 35.3)	
Annual household income, $					
<25 000	113 054	24.6 (20.4 to 28.9)	22 728	20.5 (13.3 to 27.6)	.29
25 000-49 999	153 352	33.4 (28.4 to 38.4)	37 778	26.3 (17.6 to 35.0)	
50 000-74 999	76 767	16.7 (12.9 to 20.6)	13 764	18.1 (8.7 to 27.4)	
75 000-99 999	60 784	13.3 (9.6 to 16.9)	8420	14.2 (6.4 to 22.0)	
≥100 000	54 730	11.9 (8.7 to 15.1)	14 589	26.7 (15.1 to 38.2)	
Access to medical care					
Health insurance coverage					
Private	169 764	32.1 (27.8 to 36.4)	29 753	17.6 (12.0 to 23.1)	.07
Public	345 601	65.4 (60.9 to 69.8)	77 794	23.5 (18.5 to 28.4)	
No insurance	13 457	2.5 (0.8 to 4.3)	604	4.5 (−4.5 to 13.5)	
PHP					
Does not have a PHP	38 274	7.0 (4.4 to 9.7)	2920	7.6 (0.7 to 14.6)	.01
Has a PHP	505 061	93.0 (90.3 to 95.6)	108 904	22.2 (18.3 to 26.1)	
Delay of medical care due to cost (past 12 mo)					
Has not delayed care	516 621	94.8 (93.0 to 96.7)	104 510	20.8 (17.0 to 24.6)	.40
Has delayed care	28 056	5.2 (3.3 to 7.0)	7800	27.8 (10.7 to 44.9)	
General health status					
Excellent	46 643	8.6 (5.8 to 11.3)	4915	10.5 (2.5 to 18.6)	.10
Very good	122 497	22.5 (18.6 to 26.4)	32 151	26.9 (16.8 to 36.9)	
Good	194 795	35.8 (31.4 to 40.2)	34 796	18.0 (13.0 to 23.0)	
Fair	121 719	22.4 (18.3 to 26.5)	24 038	21.3 (14.1 to 28.6)	
Poor	57 904	10.7 (7.9 to 13.4)	15 861	27.7 (16.0 to 39.5)	
**Lung cancer risk factors**					
Age, y					
55-64	295 730	54.2 (49.6 to 58.8)	47 725	16.4 (11.5 to 21.2)	.001
65-77	233 274	42.8 (38.2 to 47.4)	63 511	28.5 (22.6 to 34.4)	
78-79^c^	16 308	3.0 (1.4 to 4.5)	1162	7.2 (−1.0 to 15.4)	
Smoking status					
Former	248 020	45.5 (40.9 to 50.1)	61 576	25.3 (20.0 to 30.5)	.05
Current	297 293	54.5 (49.9 to 59.1)	50 823	17.7 (12.5 to 22.9)	
Smoking history, pack-years					
30-39	171 157	31.4 (27.1 to 35.7)	30,22	18.0 (11.7 to 24.3)	.20
40-49	179 140	32.9 (28.7 to 37.0)	34 085	19.2 (13.6 to 24.8)	
50-59	90 560	16.6 (12.6 to 20.7)	24 412	29.8 (17.6 to 42.0)	
≥60	104 456	19.2 (15.6 to 22.7)	23 674	22.9 (15.4 to 30.3)	
Years since quitting smoking^d^					
<5	73 540	29.7 (24.7 to 35.0)	20 600	28.8 (19.4 to 38.2)	.17
5-10	92 250	37.2 (31.7 to 42.9)	26 379	28.9 (19.8 to 38.0)	
11-15	82 231	33.2 (27.7 to 38.9)	14 598	18.0 (9.5 to 26.5)	

Abbreviations: GED, General Education Development; LCS, lung cancer screening; NA, not applicable; PHP, primary health professional.

^a^ All *P* values are from adjusted Wald χ^2^ tests.

^b^ Race and ethnicity are reported as collected within the Behavioral Risk Factor Surveillance System. This information was included as a variable because of disparities in LCS and outcomes. The other category includes American Indian or Alaska Native, Asian, Native Hawaiian or Pacific Islander, or race not included in the surveillance system.

^c^ Respondent age older than 80 years is collapsed within the data set; thus, we limited the sample to respondents 79 years or younger.

^d^ Only includes respondents who reported not smoking currently.

Multivariable hierarchical logistic regression analysis (Table 2) showed a higher likelihood of LCS for respondents in Rhode Island compared with Maine (OR, 1.96 [95% CI, 1.05-3.67]; *P* = .03). Compared with White respondents, the likelihood of LCS was higher for those who self-reported belonging to racial and ethnic groups other than White, Black, Hispanic, or multiracial (OR, 8.89 [95% CI, 1.81-43.71]; *P* = .01). Respondents who reported having a primary health professional (PHP) had a higher likelihood of LCS compared with those without a PHP (OR, 5.62 [95% CI, 1.19-26.49]; *P* = .03). Compared with respondents aged 65 to 77 years, the likelihood of obtaining LCS was lower for those aged 55 to 64 years (OR, 0.43 [95% CI. 0.23-0.81]; *P* = .01) and those aged 78 to 79 years (OR, 0.17 [95% CI, 0.04-0.80]; *P* = .02).

**Table 2.** Factors Associated With Obtaining LCS Among Eligible Individuals

Characteristic	OR (95% CI)	Pseudo *R*^2^	*P* value
State			
Intercept	0.06 (0.01-0.40)	0.02	.001
Maine	1 [Reference]		NA
Michigan	1.35 (0.82-2.21)		.24
New Jersey	1.02 (0.52-2.03)		.95
Rhode Island	1.96 (1.05-3.67)		.03
Demographic characteristics			
Sex			
Male	1 [Reference]	0.07	NA
Female	0.96 (0.58-1.59)		.87
Race and ethnicity^a^			
White	1 [Reference]		NA
Black	0.83 (0.23-2.99)		.78
Hispanic	0.79 (0.15-4.03)		.77
Multiracial	0.73 (0.07-7.33)		.79
Other	8.89 (1.81-43.71)		.01
Educational attainment			
4 y college or more	1 [Reference]		NA
Less than high school diploma	1.58 (0.52-4.80)		.42
High school diploma or GED	1.11 (0.56-2.23)		.76
Some college or technical school	1.61 (0.82-3.16)		.16
Annual household income, $			
100 000 or more	1 [Reference]		NA
<25 000	0.47 (0.18-1.27)		.14
25 000-49 999	0.77 (0.33-1.83)		.56
50 000-74 999	0.48 (0.19-1.23)		.13
75 000-99 999	0.45 (0.18-1.12)		.09
Access to medical care			
Health insurance coverage			
Private	1 [Reference]	0.10	NA
Public	0.81 (0.42-1.56)		.53
No insurance	0.69 (0.06-8.08)		.77
PHP			
Does not have PHP	1 [Reference]		NA
Has PHP	5.62 (1.19-26.49)		.03
Delay of medical care due to cost			
Has not delayed care	1 [Reference]		NA
Has delayed care	1.64 (0.52-5.18)		.40
General health status			
Excellent	1 [Reference]	0.12	NA
Very good	2.51 (0.81-7.77)		.11
Good	1.67 (0.57-4.92)		.35
Fair	2.60 (0.85-7.93)		.09
Poor	2.88 (0.85-9.77)		.09
Lung cancer risk factors			
Age, y		0.17	
65-77	1 [Reference]		NA
55-64	0.43 (0.23-0.81)		.01
78-79^b^	0.17 (0.04-0.80)		.02
Smoking status			
Former	1 [Reference]		NA
Current	0.61 (0.37-1.00)		.05
Smoking history, pack-years			
30-39	1 [Reference]		NA
40-49	0.84 (0.43-1.61)		.59
50-59	1.08 (0.48-2.40)		.86
≥60	1.03 (0.50-2.10)		.94

Abbreviations: GED, General Education Development; NA, not applicable; OR, odds ratio; PHP, primary health professional.

^a^ Race and ethnicity are reported as collected within the Behavioral Risk Factor Surveillance System. This information was included as a variable because of disparities in LCS and outcomes. The other category includes American Indian or Alaska Native, Asian, Native Hawaiian or Pacific Islander, or race not included in the surveillance system.

^b^ Respondent age older than 80 years is collapsed within the data set; therefore, we limited the sample to respondents 79 years or younger.

## Discussion

The prevalence of LCS in 2021 rebounded to higher levels than in 2019. However, LCS rates remain lower than other population-level cancer screening programs.^5^ Consistent with prior studies, LCS uptake varied between states. Having a PHP was associated with higher likelihood of LCS, and our findings highlight the potential importance of Medicare coverage for LCS. Limitations of this analysis include use of self-reported data that were collected in only 4 states.

Disparities in LCS uptake among eligible adults remain and will likely continue with the updated USPSTF recommendation increases the number of Black adults who are eligible for LCS.^5^ Research to identify facilitators for LCS among persons who currently smoke is needed, including a focus on the role of stigma as a barrier to screening.^5,6^ Finally, the high percentage of LCS in persons who reported poor health is concerning as many may not be healthy enough for treatment of lung cancer.

## Notes

Supplement 1. **eMethods**.
eReferences

Supplement 2.
Data Sharing Statement
