# Supplementary material for: Prevalence of Lung Cancer Screening Among Eligible Adults in 4 US States in 2021
Source: JAMA Netw Open. 2023 Jun 21;6(6):e2319172. doi: 10.1001/jamanetworkopen.2023.19172 (PMC10285576; doi:10.1001/jamanetworkopen.2023.19172)
Supplement: Supplement 1. — eMethods. eReferences [file jamanetwopen-e2319172-s001.pdf]

Supplemental Online Content

Maki KG, Tan NQP, Toumazis I, Volk RJ. Prevalence of lung cancer screening among eligible adults in 4 US states in 2021. *JAMA Netw Open.* 2023;6(6):e2319172. doi:10.1001/jamanetworkopen.2023.19172

eMethods.
eReferences

This supplemental material has been provided by the authors to give readers additional information about their work.

© 2023 Maki KG et al. *JAMA Network Open.*

eMethods.

Data Source

We used data from the public-use 2021 Behavioral Risk Factor Surveillance System (BRFSS),[1] which is conducted by the Population Health Surveillance Branch of the Centers for Disease Control and Prevention (CDC). The purpose of the BRFSS is to collect health-related data from noninstitutionalized adults in the United States and participating areas (e.g., District of Columbia and participating U.S. territories).

The data were collected with telephone surveys, with both landline and cellphone numbers in the United States and participating areas. Responses are self-reported and proxy interviews are not reported. For landline interviews, data are collected from a randomly selected adult in the household. For cellphone interviews, data are collected from the individual who answered the cellphone; responses are limited to adults residing in a private residence or college housing. The interviews for the 2021 BRFSS were conducted from January 3, 2021, through February 28, 2022. Data on lung cancer screening came from the optional lung cancer screening module of the BRFSS, which were used by 4 states in 2021 (Maine, Michigan, New Jersey and Rhode Island).

The content of the BRFSS is agreed upon by the BRFSS coordinators and the CDC. There is a core component in the questionnaire, along with optional modules, and state-added questions. All states must ask the core component questions without modification. The states may include any, all, or none of the optional modules and they may also add questions for their state. Complete details about the data collection process are in the BRFSS Data and Documentation materials available online.[1]

Measures

The outcome variable was derived from the following question, “In the last 12 months, did you have a CT or CAT scan?” with response options including, “Yes, to check for lung cancer;” “No (did not have a CT scan);” “Had a CT scan, but for some other reason;” and “Don’t

© 2023 Maki KG et al. *JAMA Network Open.*

know/Not sure." For our analysis, we dichotomized the variable to: 1 = "Yes, to check for lung cancer" response; 0 = "No (did not have a CT scan)" or "Had a CT scan, but for some other reason"; "Don't know/Not sure" was coded as missing.

The independent variables included sociodemographic items, access to care (health insurance, having a primary health professional, and delay of medical treatment in the past year due to cost). Smoking history variables were calculated as shown in eTable1.

eTable 1. Calculation of smoking history variables

**Input Variable(s), Question asked**	**Calculation**	**Calculated variable, Description**	**Final Variable**
LCSNUMCG<br><br>On average, when you smoke/smoked regularly, about how many cigarettes do/did you usually smoke each day?	LCSNUMCG/20	PacksDay<br><br>Packs of cigarettes smoked per day	PackYears
LCSLAST<br><br>How old were you when you last smoked cigarettes regularly?<br><br>LCSFIRST<br><br>How old were you when you first started to smoke cigarettes regularly?	LCSLAST-LCSFIRST	YearsSmoked<br><br>Number of smoking years	
PacksDay<br><br>YearsSmoked	PacksDay*YearsSmoked	PackYears<br><br>Estimated number of smoking pack-years	
SMOKDAY2<br><br>Do you now smoke cigarettes every day, some days, or not at all?<br><br>@_AGE80	IF(SMOKDAY2=3)<br><br>YearsQuit=@_AGE80-<br><br>LCSLAST	YearsQuit<br><br>Estimated number of years since quitting smoking	YearsQuit

© 2023 Maki KG et al. *JAMA Network Open.*

Imputed age value, collapsed above 80<br><br>LCSLAST<br><br>How old were you when you last smoked cigarettes regularly?			

Analysis Plan

Our sample (weighted N = 545,313) included respondents who met the following criteria: 1) were between 55 to 79 years of age; and 2) reported a smoking history of ≥ 30 pack-years and currently smoked or had quit within 15 years. We excluded respondents who reported a history of lung cancer (eFigure 1). We used the 2013 USPSTF recommendation criteria on LCS criteria because the Centers for Medicare and Medicaid Services coverage determination was not updated until February 2022[2] and private insurers coverage updates have varied with the 1 year grace period allowed.[3]

eFigure 1. Study population of respondents eligible for lung cancer screening

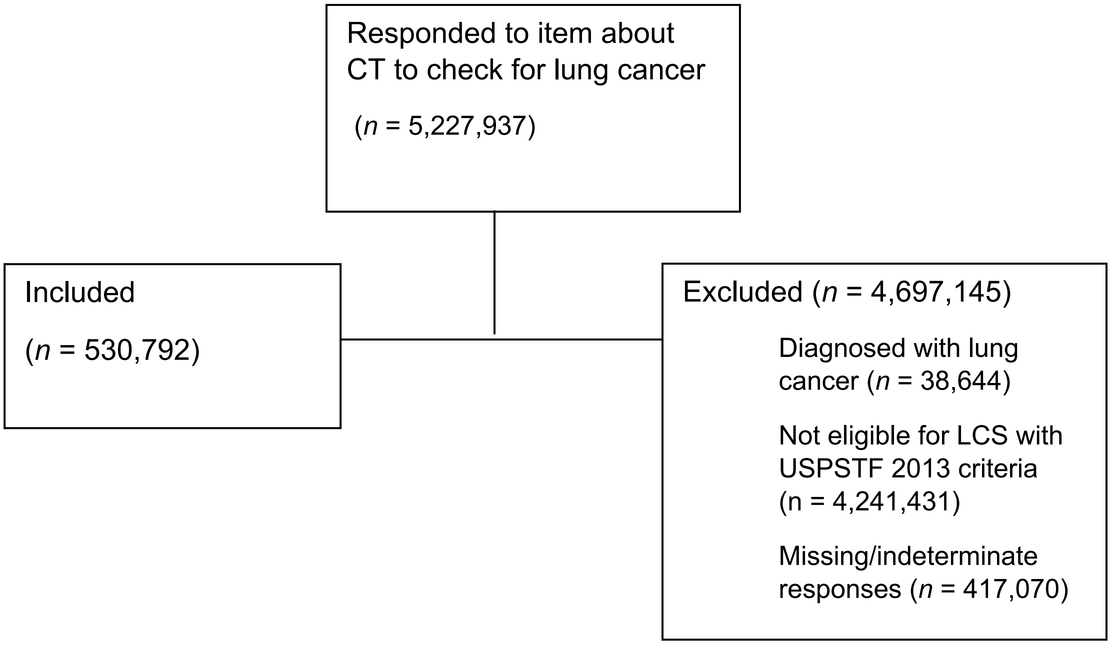

© 2023 Maki KG et al. *JAMA Network Open.*

Although our sample had a lower percent of missing data (17.6%) for the income variable than the full dataset (21.5%), we acknowledge there could be bias due to missing data. We used listwise deletion for our analysis. For the multivariable hierarchical logistic regression analysis, we did not include years since quit (YSQ) in the model due to using listwise deletion (this would have excluded respondents who were currently smoking). A full summary of the variables used in the analysis and the percent missing is included in eTable 2.

eTable 2. Variables used in analysis

Variable	Description	unweighted *n*	%	weighted *n*	%
**Outcome**					
HadLCS	Recode of LCSCTSCN where yes to check for lung cancer = 1, no = 0				
0	No	1033	75.5	418393	78.8
1	Yes	308	22.5	112399	21.2
7, 9	Missing/not applicable	28	2.3		
**Additional variables**					
RaceEth	Recode of RACEGR3 to assign NH-White as referent				
0	NH White only	1210	88.4	467654	89.1
1	NH Black	30	2.2	24247	4.6
2	Hispanic	25	1.8	15089	2.9
3	Multiracial	26	1.9	3087	0.6
4	All other single and multiple groups	46	3.4	14516	2.8
9	Missing	32	2.3		
Female	Recode of SEXVAR where male=0 and female=1				
0	Male	708	51.7	290460	53.3
1	Female	661	48.3	254853	46.7
9	Missing	0	0		
Edu	Recode of EDUCA				
0	4-year college or higher	293	21.4	58118	10.7
1	Less than high school diploma/GED	122	8.9	84366	15.5
2	High school/GED or equivalent	534	39.0	229878	42.2
3	Some college	419	30.6	172585	31.7
9	Missing	1	0.1		
IncGroups_rc	Recode of INCOME3				
0	$100,000 and more	119	8.7	54730	11.9
1	<$25,000	327	23.9	113054	24.6

© 2023 Maki KG et al. *JAMA Network Open.*

2	$25,000 to $49,999	383	28.0	153352	33.4
3	$50,000 to $74,999	177	12.9	76767	16.7
4	$75,000 to $99,999	124	9.1	60784	13.3
9	Missing	239	17.5		
STATE	State of residence				
23	Maine	603	44.0	52002	9.5
26	Michigan	386	28.2	299670	55.0
34	New Jersey	184	13.4	169728	31.1
44	Rhode Island	196	14.3	23913	4.4
99	Missing	0	0		
InsType	Recode of PRIMINSR				
1	Private	354	25.9	169764	32.1
2	Public	948	69.2	345601	65.4
3	No insurance	32	2.3	13457	2.5
9	Missing	35	2.6		
HasPCP	Recode of PERSDOC3 to create dichotomous variable				
0	Does not have regular Dr	71	5.2	38274	7.0
1	Yes, has one or more	1290	94.2	505061	93.0
9	Missing	8	0.6		
MedCost	Recode of MEDCOST1				
0	Has not delayed	1289	94.2	516621	94.8
1	Has delayed	78	5.7	28056	5.2
7	Missing	2	0.1		
GENHLTH	General health				
1	Excellent	90	6.6	46643	8.6
2	Very good	323	23.6	122497	22.5
3	Good	500	36.5	194795	35.8
4	Fair	313	22.9	121719	22.4
5	Poor	139	10.2	57904	10.7
7,9,99	Missing/refused	4	0.3		
SmkCurrent	Recode of SMOKDAY2 to make dichotomous variable				
0	Do not currently smoke	701	51.2	248020	45.5
1	Currently smoke	668	48.8	297293	54.5
7,9,99	Missing	0	0		
PY_Groups	Recode of PackYears				
2	30 to 39	387	27.6	171157	31.4
3	40 to 49	471	34.4	179140	32.9
4	50 to 59	221	16.1	90560	16.6
5	60 to highest	299	21.8	104456	19.2
99	Missing	0	0		
YSQ_Groups	Recode of YearsQuit				
0	Lowest to 4	224	16.4	73540	29.7
1	5 to 10	251	18.3	92250	37.2

© 2023 Maki KG et al. *JAMA Network Open.*

2	10 to 15	226	16.5	82231	33.2
99	Missing/not applicable	668	48.8		
Age_Medicare_rc	Recode of @_AGE80				
0	65 to 77	687	50.2	233274	42.8
1	55 to 64	645	47.1	295730	54.2
2	78 to 79	37	2.7	16308	3.0
9	Missing	0	0		
Missing		51	2.0		
Mort_5YrGroups_rc	Recode for groups in 5-year mortality % estimates				
5	5	340	13.2	2698170	15.8
8	8	648	25.2	4549366	26.6
12	12	570	22.2	3716451	21.8
19	19	329	12.8	2185229	12.8
30	29-37	410	15.9	2538478	14.9
50	49-62 (highest)	225	8.7	1385197	8.1
99	Missing	51	2		

Note: Study sample is respondents who have not been diagnosed with lung cancer and would be eligible for lung cancer screening using the 2013 USPSTF eligibility criteria.

We used the haven[4] and survey[5] packages within RStudio[6] for this analysis. We used haven[4] to import the data to RStudio and conducted our main analysis with the survey[5] package. This allowed us to account for the complex sampling design and use the weights provided with the BRFSS data to represent the U.S. population at the time of data collection.[1]

© 2023 Maki KG et al. *JAMA Network Open.*

eReferences

1. Centers for Disease Control and Prevention. Behavioral Risk Factor Surveillance System Data & Documentation. Accessed January 23, 2023, 2023. https://www.cdc.gov/brfss/data_documentation/index.htm
2. Center for Medicare and Medicaid Services. Screening for Lung Cancer with Low Dose Computed Tomography (LDCT). Updated February 10, 2022. Accessed January 10, 2023, 2023. https://www.cms.gov/medicare-coverage-database/view/ncacal-decision-memo.aspx?proposed=N&ncaid=304
3. American College of Radiology. Status of Lung Cancer Screening Coverage. Accessed 02/13/2023, 2023. https://www.acr.org/-/media/ACR/Files/Clinical-Resources/Status-of-Lung-Cancer-Screening-Coverage.pdf
4. Wickham H, Miller, E., & Smith, D. haven: Import and Export 'SPSS,' 'Stata' and 'SAS' Files. 2023. https://github.com/tidyverse/haven
5. Lumley T. survey: analysis of complex survey samples. R package version 4.0. Accessed January 23, 2023, 2023. https://cran.r-project.org/web/packages/survey/index.html
6. Team R. RStudio: Integrated Development for R. RStudio, PBC. Accessed January 23, 2023, 2023. http://www,rstudio.com

© 2023 Maki KG et al. *JAMA Network Open.*
