# Supplementary material for: Prevalence of Lung Cancer Screening Among Eligible Adults in 4 US States in 2021
Source: JAMA Netw Open. 2023 Jun 21;6(6):e2319172. doi: 10.1001/jamanetworkopen.2023.19172 (PMC10285576; doi:10.1001/jamanetworkopen.2023.19172)
Supplement: Supplement 2. — Data Sharing Statement [file jamanetwopen-e2319172-s002.pdf]

Data Sharing Statement

Maki. Prevalence of Lung Cancer Screening Among Eligible Adults in 4 US States in 2021. *JAMA Netw Open*. Published June 21, 2023. doi:10.1001/jamanetworkopen.2023.19172

Data
Data available: Yes
Data types: Deidentified participant data
How to access data: Data are available online through BRFSS, https://www.cdc.gov/brfss/annual_data/annual_2021.html
When available: beginning date: 02-17-2023

Supporting Documents
Document types: Other (please specify)
Additional Information: Analysis plan and variables used in our study are included in the online supplement.
How to access documents: The analysis plan and variables used in our sample are included in the online supplement. The data are readily available on the BRFSS website.
When available: With publication

Additional Information
Who can access the data: The online supplement will be available to anyone reading the article who accesses the link.
Types of analyses: The analysis plan we used and variables (including how variables were recoded) is included in the supplement.
Mechanisms of data availability: This is included in the supplement.
